# Prevalence of Sexual Strangulation/Choking Among Australian 18–35 Year-Olds

**DOI:** 10.1007/s10508-024-02937-y

**Published:** 2024-07-03

**Authors:** Leah S. Sharman, Robin Fitzgerald, Heather Douglas

**Affiliations:** 1 Melbourne Law School, Faculty of Law, University of Melbourne, Melbourne, VIC 3010 Australia; 2 Faculty of Humanities and Social Science, University of Queensland, Brisbane, Australia

**Keywords:** Choking, Strangulation, Asphyxiation, Breath play, Transgender, Sexual orientation

## Abstract

In Australia, strangulation has been explicitly criminalized in all states and territories. However, it continues to be a “normalized” sexual practice despite its potentially fatal consequences and associated short and long-term sequelae. This research aimed to establish the prevalence of strangulation during sex and examine predictors of positive perceptions toward sexual strangulation in Australia. Confidential, cross-sectional online surveys were conducted with 4702 Australians aged 18–35 years. Participants were 47% cis-men, 48% cis-women, and 4% trans or gender diverse. A total of 57% reported ever being sexually strangled (61% women, 43% men, 79% trans or gender diverse) and 51% reported ever strangling a partner (40% women, 59% men, 74% trans or gender diverse). Differences were found across genders on all variables of sexual strangulation, including frequency of engagement, level of pressure on the neck, consequences, wanting and enjoyment, and how consent was given/received. However, when split by gender, sexual orientation of men and women revealed further differences in behaviors, consequences, and wanting, particularly among straight and bisexual women. After accounting for exposure to strangulation in pornography and previous experience of sexual strangulation, positive perceptions of being strangled (*R*^2^ = .51) and strangling a partner (*R*^2^ = .53) were predicted by ratings that it could be done safely and social normative factors. These findings suggest strangulation is common during sex among young Australians. Non-stigmatizing education strategies are needed to engage with young people so they have a better understanding of the risks involved and how to negotiate consent and safety regarding sexual strangulation.

**Supplementary Information:**

The online version contains supplementary material available at 10.1007/s10508-024-02937-y.

## Introduction

Recognition of the dangers of strangulation as part of domestic violence has led to the introduction of offences in all states and territories in Australia (Douglas, 2023) and in several other countries (Edwards & Douglas, 2021). However, research from the US (Herbenick et al., 2020, 2021, 2022a), and preliminary investigations in New Zealand (Beres et al., 2020) and Australia (Sharman et al., 2024) show that strangulation also occurs beyond the context of domestic violence as part of sexual activity, and particularly among adults younger than 40 (Herbenick et al., 2023a; Pavanello et al., 2024; Vilhjálmsdóttir & Forberg, 2023).

More commonly known as “choking,” strangulation of the neck during sex refers to a range of behaviors that restrict the flow of breath or blood. This can include the use of hands, arms (e.g., chokehold), feet, and ligatures such as belts, ropes, and ties. Although signs of injuries are often not externally visible, the consequences can range from a sore throat and bruising to neurological damage, unconsciousness, and even death (Huibregtse et al., 2022; Sharman et al., 2023). Given the potential for significant harm and lethality associated with this type of asphyxiation, “strangulation” has at times been a preferred term to acknowledge its specific risks compared with “choking” that may minimize its potential harm, particularly in unequal relationships where there is violence (Busby, 2012; Glass et al., 2008). However, strangulation is often perceived to be meaningfully different in its intention, as a more violent act, compared to choking, which may be viewed as part of intimacy (Beres et al., 2020; Herbenick et al., 2022b). The difference between what is understood as choking compared to strangulation are not always consistent across individuals, but does, so far, appear to be a continuum of how safety is perceived (Beres et al., 2020; Herbenick et al., 2022b). For example, it has been described as using one (choking) vs two hands (strangulation) or choking as safe and strangulation as unsafe or related to death (Herbenick et al., 2022b). In this paper, we use “strangulation” or “sexual strangulation” to acknowledge its potential for significant harm as well as medical accuracy in describing the activity of external compression of the neck, rather than “choking” which involves the partial or total obstruction of the trachea by a foreign substance (Glass et al., 2008; White et al., 2021). However, we use “choking” where relevant to reflect the language captured in our data and used by the general population.

While the risk of injury is substantial for strangulation, the type and severity of consequences may differ depending on the context (e.g., consenting sex versus domestic violence), individual characteristics of the person being strangled (e.g., amount of physical pressure they can tolerate, and number of times they have been strangled), and duration of anoxia/hypoxia (Dunn et al., 2023). Physical consequences can be significant and injuries long lasting, developing weeks or months after the strangulation, particularly following multiple strangulations (Huibregtse et al., 2022). However, some people who are strangled report experiencing pleasure more commonly than negative consequences, although the same physical experiences and alterations of consciousness can be perceived as positive or negative by different people and with different sexual partners (Dunkley et al., 2020; Herbenick et al., 2022b, 2022c). While the dangers of engaging in strangulation are well-established, previous research involving 167 Australian undergraduate students found that young adults who engage in strangulation during sex often feel that it could be safe and are generally unaware of when injuries may develop (Sharman et al., 2024).

Although strangulation is typically gendered, with women more often strangled and men more likely to be the strangler (Herbenick et al., 2021, 2022a, 2022b, 2022c; Vilhjálmsdóttir & Forberg, 2023), there is still similarity across men and women in their rates of ever engaging in either activity that are only partially explained by sexual orientation (Herbenick et al., 2021, 2022a). Research suggests that pornography and other media, including memes and magazine articles that can be accessed online, are helping to drive increased awareness and willingness to engage in strangulation, as well as young people’s belief that it is a safe practice (Herbenick et al., 2020, 2022c, 2023b, 2023c; Wright et al., 2023a). An analysis of pornography use among young men found that watching pornography was associated with a higher likelihood of being exposed to depictions of strangulation during sex, which in turn predicted a higher likelihood of strangling sexual partners (Wright et al., 2023a). However, this relationship was mediated by the belief that the act was pleasurable, safe, and that it did not require consent. These results are echoed in a qualitative study where most women interviewed who had been strangled/choked during sex reported that it often happened without explicit consent or where their partners assumed their consent (Herbenick et al., 2022b). Similarly, interviewed males who had strangled/choked partners reiterated that in ongoing relationships consent to sexual strangulation is often only discussed once, and future consent is commonly assumed rather than actively sought from partners’ (Herbenick et al., 2022c). Recent quantitative research from Iceland also revealed that 27.3% of 227 participants reported that strangulation/choking occurred without consent being obtained prior to the act (Vilhjálmsdóttir & Forberg, 2023). This belief that strangulation is safe and therefore does not require consent within each new event of sexual strangulation, alongside limited knowledge about its potentially dangerous consequences, paint a concerning picture about its uptake in the general population. This is particularly so given evidence that women may sometimes opt for submissive roles to please partners (Bridges et al., 2016; Herbenick et al., 2022b).

Perceptions of sexual strangulation in Australia among a sample of Queensland undergraduates appeared to generally show that it is perceived positively, and this perception was associated with more frequent sexual strangulation both in the role of the strangled and strangler (Sharman et al., 2024). From the literature explored here there appear to be a number of potential reasons why sexual strangulation is perceived positively, including beliefs that it is safe and pleasurable as presented in depictions of strangulation in pornography (Wright et al., 2023a). Broader research on risky sexual behaviors may also help to explain some of these perceptions. In particular, research findings that show when their peers are perceived to take more sexual risks, university-aged adults tended to engage in more sexually risky behaviors (e.g., sex with someone they just met, sex without a condom; Lewis et al., 2007; Winslow et al., 1992). It is possible, then, that views of sexual strangulation as a “safe” and socially normative activity, help to predict more positive perceptions of strangulation during sex.

Unlike the US, the national prevalence of sexual strangulation is not known in Australia, and we know little about how it is learned, the demographic characteristics of who is engaging in it, how consent is given or understood, and the level of force used on the neck. Thus, the primary aim of this research was to provide a first national picture of sexual strangulation awareness, engagement, practices, and acceptance among young Australian adults (18–35 years) by gender and sexual orientation. Secondly, we aimed to understand what predicts positive perceptions of sexual strangulation outside of pornography and prior experience. We hypothesized that greater positive perceptions of sexual strangulation would be explained by social and safety factors. That is, when accounting for prior exposure to sexual strangulation in pornography and engagement in sexual strangulation, beliefs that sexual strangulation can be safe, perceptions that sexual strangulation is now a normative part of sex and having discussed it with peers, would individually and in combination predict greater positive perceptions of being strangled and strangling a partner.

## Method

Survey questions assessed here were part of a larger study on sexual strangulation. Questions were utilized from a previous study of undergraduate students (Sharman et al., 2024). The current survey is a refinement of the earlier questions that had been previously piloted and tested among undergraduate students. Questions for this larger survey were refined by the authors based on feedback and results from the earlier survey. Further adjustments were made after discussion and advice from sexual health educators with extensive knowledge on sexual strangulation among adolescents and young adults, and who had completed independent interviews with young people on their strangulation experiences during sex. Given the common usage of the term “choking,” all questions used “choking” instead of “strangulation” and are referred to a such in the results.

### Participants and Procedure

The sample included 4702 volunteers from the Australian general public aged 18–35 years (*M* = 27.30, *SD* = 4.73). From the full sample (*N* = 5071), 352 participants were excluded because they had not previously had a “sexual experience,” which was left undefined for participants to freely interpret; 17 were removed for incongruency between quantitative survey responses indicating experiences of “choking” and qualitative responses indicating none, or misunderstanding “choking” to include body parts other than the neck; and one was removed due to duplication.

The survey was administered through the Online Research Unit (ORU; https://www.theoru.com), who recruited participants and distributed the survey through established voluntary research panels. Participants who were previously identified by the ORU as being aged 18–35 were recruited via emails about “a new online survey” with a guide for the approximate maximum length of the survey (15 min), and remuneration. The link then took them directly to the information sheet and consent to participate. However, the email did not identify any information about the research, only the availability of a survey for participation. Participants were reminded at three informational intervals about the anonymity of their responses, and that they could skip any questions or exit the survey if they no longer wished to participate. Links to services were provided in case participants felt any distress answering questions. Stratified sampling was used by the ORU to gain a demographically representative national sample for age, gender, and location, with that information provided by potential participants on sign-up to the voluntary panels. Targeted sampling of non-binary, and transgender participants was carried out for this study to ensure these groups could be adequately represented in the study. This occurred through early invitations to the survey to panelists who had previously identified at sign-up to ORU as non-binary, transgender, or gender questioning before sampling more widely across the panels. This method ensured that these groups had greater opportunity to participate before the survey closed. The survey was open for over 52 days until a minimum of 5000 responses were reached. Participants were awarded points worth a nominal value ($2.00) upon completion of the survey. Response rate for the survey was 20% via the emailed advertisement of “a new online survey” with an 86% completion rate.

### Measures

Except for gender, location, and age, participants were able to select “prefer not to answer” for all survey questions, including when separately asked whether they identified as transgender.

#### Demographics

Participants were asked demographic questions of their gender, sexual orientation, relationship status, education status, and ethnicity to assess their similarity to the general population. These are provided in Table 1. Overall, the demographics of respondents were similar to that of the general population (Australian Institute of Health & Welfare, 2021), with sexuality and gender more diverse (Wilson et al., 2020) in line with our oversampling of non-binary, transgender and gender questioning participants.

**Table 1:** Participant demographics

		Total (*N* = 4702)	Men (*N* = 2228)	Women (*N* = 2293)	TGD (*N* = 181)
Trans and gender diverse (TGD)		3.8% (181)	43.1% (78)	26% (47)	31% (56)
*Sexuality*					
Straight		81.9% (3853)	88.6% (1975)	79.9% (1831)	26% (47)
Gay or Lesbian		4.2% (196)	5% (111)	2.4% (55)	16.6% (30)
Bisexual		9.7% (455)	4.3% (96)	13.1% (301)	32% (58)
Pansexual		1.9% (87)	.5% (11)	2% (46)	16.6% (30)
Asexual		0.6% (29)	< 10	0.7% (16)	< 10
Other		0.4% (18)	< 10	< 10	< 10
*Last sexual activity*					
Man		50.6% (2377)	19.4% (433)	81.9% (1878)	36.5% (66)
Woman		47.7% (2241)	79.6% (1774)	16.7% (383)	46.4% (84)
TGD		0.9% (44)	< 10	< 10	15.5% (28)
Other (e.g., threesome)		< 10	–	–	–
*Relationship status*					
Single		32.6% (1534)	37% (825)	27.6% (634)	41.4% (75)
In a relationship (not living together)		13.3% (625)	12.7% (283)	13.8% (316)	14.4% (26)
In a relationship (living together/Defacto)		27.6% (1296)	24.3% (541)	31% (711)	24.3% (44)
Married		25% (1177)	25% (556	25.6% (588)	18.2% (33)
Divorced/Separated		1% (45)	0.6% (13)	1.3% (30)	< 10
Widowed		< 10	–	–	–
Other		< 10	–	–	–
*Education*					
Did not complete final year of high school		5.7% (269)	4.8% (106)	6.5% (150)	7.2% (13)
Completed final year of high school		20.8% (977)	20.7% (461)	20.2% (464)	28.7% (52)
Certificate or Diploma		26.2% (1230)	496 (22.3%)	30% (688)	25.4% (46)
Undergraduate degree		33.1% (1556)	36.4% (810)	30.6% (702)	24.3% (44)
Masters degree		12.4% (582)	13.6% (304)	11.1% (255)	12.7% (23)
Doctoral degree		1.3% (63)	1.8% (41)	0.9% (20)	< 10
*Ethnicity*					
White/European		65.7% (3091)	63.2% (1402)	68.8% (1578)	58.6% (106)
Aboriginal		4.8% (226)	4.2% (94)	0.2% (115)	9.4% (17)
Torres Straight Islander		0.3% (15)	< 10	< 10	< 10
Pacific Islander		1.7% (80)	1.8% (41)	1.4% (33)	3.3% (6)
East Asian		4.3% (201)	5.5% (122)	3.1% (71)	< 10
South Asian		6.3% (296)	7.8% (173)	4.6% (106)	9.4% (17)
Southeast Asian		7.3% (343)	8.2% (182)	6.8% (155)	< 10
Central Asian		1.6% (75)	1.3% (30)	2% (45)	< 10
Middle Eastern		1.9% (91)	1.9% (42)	2% (46)	< 10
Black/African or African American		1.3% (60)	1.7% (37)	0.9% (21)	< 10
Spanish/Latin American		1.1% (51)	1.3% (30)	0.9% (21)	–
Mixed ethnicity		1.9% (88)	1.4% (32)	2.3% (52)	< 10
Other		.5% (23)	< 10	0.6% (13)	< 10

#### Gender and Sexual Orientation

All participants were asked about their gender (man, woman, non-binary, questioning/unsure, additional gender category/identity not specified) and sexual orientation (heterosexual [straight], homosexual [gay/lesbian], bisexual, pansexual, asexual, additional category/identity not listed). A free text entry area was available to specify further if they wished. Participants were separately asked if they identified as transgender (trans). These were grouped according to sample sizes for analysis. Gender was coded into three categories, 1 = *cisgender men,* 2 = *cisgender women*, and 3 = *transgender and gender diverse* (trans identifying, non-binary, questioning/unsure, additional category). Sexual orientation was categorized as *straight* (1), *gay/lesbian* (2), and *bisexual* (3). Pansexual, asexual, and additional sexualities were not utilized in separate analyses due to low sample sizes.

#### Peers and Awareness of Choking

Participants were asked whether they had ever discussed sexual choking with their friends and asked to select any of the responses that applied. Two of these items related to being choked (e.g., “Yes, among my same gendered friends, we have discussed being choked”) and two related to choking others (e.g., “Yes, among my friends of different genders we have discussed choking sexual partners”). Answers were combined into two variables for peer discussions about being choked and choking a partner. These were binary coded for analysis *no* (0) and *yes* (1).

Participants were asked separately at what age they first learned about choking as *15 or under* (1), *16–18* (2), *19–21* (3), *22–24* (4), *25–30* (5), *30–35* (6), and *unsure* (96). They were also asked where they had first learned about sexual choking, and where else they had ever seen or heard about sexual choking. For both questions, participants were presented with a list of options (e.g., “pornography,” “erotica,” “social media”; see supplementary material, Table S1, for full list) that were binary coded as *no* (0) or *yes* (1). Answers to both questions were combined to understand where participants had “ever” been exposed to sexual choking. Ever having seen sexual choking in pornography was a binary variable used in regression analyses.

#### Perceptions of Choking

All participants were asked to rate their agreement with the statement “I think choking can be done safely,” and a statement about their expectation of choking as part of sexual experiences* “*Choking is an expected part of sex*.”* Participants were also asked to rate their agreement on positive personal perceptions of choking across four items on being choked. If participants had not experienced this before, they were asked to answer how they think they would feel (e.g., “I would enjoy being choked during sex” or “Being choked during sex would make me feel excited”) and the same items worded for positive perceptions of choking a partner (e.g., “I would enjoy choking my partner during sex”). All statements were rated on a 5-point agreement scale from *strongly disagree* (1) to *strongly agree* (5). These items have been used previously (Sharman et al., 2024). However, for both scales removing the reverse scored item “Being choked/choking my partner during sex would make me feel afraid or fearful” substantially improved the reliability of the scale from ⍺_choked_ = 0.78–0.86, respectively, and ⍺_choking_ = 0.74–0.89, respectively, and were therefore removed for the purposes of analyses.

#### Experiences of Choking

Personal experiences of sexual choking were asked of participants who had been choked or choked a partner using mirrored items. Participants who identified that they had been choked (see below) by a partner saw wording related to being choked and those who had choked a partner saw wording related to having choked a partner during sex. Participants saw both sets of questions if they had both been choked and choked a partner.

#### Ever Participated in Choking

All participants were asked if they had ever experienced any of the following during sex: “Had your partner’s hands on or around your neck/throat,” “Been the submissive in breath-play,” “Felt your neck/ throat was being pushed or pressed,” “Had ropes or ties around your neck,” “Had difficulty breathing because of pressure my partner put on my neck.” Participants could select multiple options (coded as 1 if selected) or select “none.” The same questions were asked about choking a partner (e.g., “had your hands on or around your partner’s neck/throat”). Any of these items that were selected were respectively combined and coded into ever being choked or ever choked a partner as *no* (0) and *yes* (1). Qualitative data, in the form of open-ended responses, were not examined in this study but were checked to ensure participants who disagreed that they had been choked despite selecting yes to any of these items were excluded.

To focus on strangulation of the neck, all participants were then provided the following definition:

The following questions refer to “strangulation,” often called “choking.” Strangulation or choking is when a person’s breathing is stopped or restricted by the use of hands, other body parts, or ties (like ropes) around the neck. In future questions, we will refer to this as “choking.”

This definition was provided to align with legislation around non-fatal strangulation in different jurisdictions in Australia (Edwards & Douglas, 2021). However, this does not include the potential for strangulation/choking of carotid arteries and/or jugular veins without restricting airways, sometimes referred to as a “blood choke” (Wedlake & Rowe, 2009).

Participants who had ever participated in choking were asked “How many sexual experiences have you had where you were choked?” Responses were provided in a dropdown from “1” to “20 or more times.” Participants were also asked how many partners they had experienced this with (e.g., “how many different partners have choked you during sex?”), and at what age they were when they were first choked. Participants could answer their age *15 or under* (1), *16–18* (2), *19–21* (3), *22–24* (4), *25–30* (5), *30–35* (6), and *unsure* not analyzed. Similarly worded items were presented to participants who had ever choked a partner.

Participants who had responded yes to ever having been choked or choking a partner were also asked separately if “during your last sexual experience, were you choked?” or “… did you choke your sexual partner?” Responses were *yes* (1) *no* (2) and *maybe/don’t remember* (3). Only *yes* and *no* were analyzed.

#### The Last Time Sexual Choking Occurred

Participants were asked to think about the last time they engaged in sexual choking and asked how much they agreed, using the same 5-point scale, with statements regarding how well they knew their sexual partner (“I knew my partner very well”), how much they enjoyed it (“I enjoyed being choked/choking my partner”), how much they perceived their partner to have enjoyed it, and whether they felt it was a common experience for them to be choked or choke a partner.

Participants were asked how the person being choked consented on this occasion, with instructions detailing that consent could include “verbal or physical movements that convey agreement.” This was assessed using discrete answers and for participants who had been choked included: “I asked them to choke me,” “They asked to choke me and I agreed,” “I gave consent and then withdrew it (e.g., told them to stop or moved their hand/s away),” “In a previous sexual encounter I gave my consent to be choked in the future,” “I did not consent beforehand but I enjoyed it,” and “I did not consent beforehand and I didn’t ask or motion for them to stop.” Similarly worded questions were presented for those who choked partners (e.g., “They asked me to choke them”).

Participants were asked “How were you choked”/ “How did you choke them” with the options “one hand,” “two hands,” “belt, tie, rope, or string,” and “other” where participants could specify another method. Pressure experienced or exerted was assessed using a sliding scale from 1 = “no pressure, just resting” to 7 = “very firm or tight pressure.”

#### Consequences

Participants were asked generally “When you have previously been choked by your partner, what were the consequences, if any?” Participants could select multiple options, including “nothing happened (positive or negative)” as well as six positive/pleasurable consequences of choking (e.g., “I enjoyed sex more”) and 12 negative/physical consequences of choking (e.g., “I couldn’t move or speak”). Participants who had choked partners were asked the same questions about their perceptions of positive consequences for those partners. See supplementary material, Table S3, for all options. These were summed to form two separate scores: one for the number of positive or pleasurable effects and the other for the number of negative physical consequences. Although we acknowledge that individuals will interpret the “positive” or “negative” valence of things like inhibiting breathing, marks and bruises, and changing vision differently, we opted to label them as we have as are result of the substantial literature connecting these acts and consequences to serious injury and/or death.

### Statistical Analyses

Analyses were conducted using SPSS v29. Descriptive statistics are presented as numbers and proportions in Table 2. Chi-squared tests (*χ*^2^) and ANOVAs were used to identify group differences examined using pairwise comparisons of proportions (*z*-tests) or post hoc multiple comparisons using Bonferroni adjustments. Data were compared by gender and sexual orientation within men and women. Examinations of sexual orientation were only investigated within men and women, excluding trans and gender diverse participants due to sample sizes.

**Table 2:** Prevalence of sexual strangulation and related behaviors, consequences, and acceptance across gender and sexual orientation

		Total M (SD) or % yes of sample					Men M (SD) or % yes of sample				Women M (SD) or % yes of sample			
		Sample (*N* = 4702)	Men (*N* = 2228)	Women (*N* = 2293)	TGD (*N* = 181)	Gender *p*	Straight (*N* = 1975)	Gay (*N* = 111)	Bisexual (*N* = 96)	Men *p*	Straight (*N* = 1831)	Lesbian (*N* = 55)	Bisexual (*N* = 301)	Women *p*
	Ever seen in porn^i^	61.3% (2880)	71.4% (1591)^a^	25.1% (2293)^b^	60.2% (109)^c^	** < .001**	71.1% (1405)^a^	85.6% (95)^b^	69.8% (67)^a^	**.004**	48.5% (888)^a^	61.8% (34)^a,b^	67.4% (203)^b^	** < .001**
	Ever discussed being strangled with peers^i^	29% (1362)	71.4%(1591)^a^	51.5% (1180)^b^	60.2% (109)^c^	** < .001**	21.1% (417)^a^	38.7% (43)^b^	38.5% (37)^b^	** < .001**	29.4% (539)^a^	41.8% (23)^b^	48.5% (146)^b^	** < .001**
	Ever discussed strangling partners with peers^i^	29.1% (1370)	31.6% (705)^a^	25% (574)^b^	50.3% (91)^c^	** < .001**	30.9% (611)^a^	36% (40)^a,b^	45.8% (44)^b^	**.006**	21.6% (1435)^a^	32.7% (18)^a,b^	44.2% (133)^b^	** < .001**
	Perceptions of being strangled	2.58 (0.98)	2.55 (0.93)^a^	2.56 (1.02)^a^	3.05 (0.80)^b^	** < .001**	2.53 (0.92)	2.70 (0.97)	2.74 (1.03)	.025*	2.48 (1.02)^a^	2.68 (0.99)^a,b^	3.01 (0.96)^b^	** < .001**
	Perceptions of strangling	2.55 (0.95)	2.70 (0.94)^a^	2.37 (0.93)^b^	3.00 (0.92)^c^	** < .001**	2.69 (0.93)	2.73 (1.05)	2.72 (1.06)	.886	2.30 (0.91)^a^	2.65 (1.01)^b^	2.69 (0.96)^b^	** < .001**
	Strangulation can be safe	3.68 (1.28)	3.67 (1.23)^a^	3.67 (1.34)^a^	3.96 (1.17)^b^	**.015**	3.56 (1.23)^a^	3.99 (1.10)^b^	3.71 (1.30)^a,b^	**.022**	3.55 (1.37)^a^	4.08 (1.04)^b^	4.18 (1.07)^b^	** < .001**
	Strangulation is expected	2.14 (1.23)	2.32 (1.26)^a^	1.91 (1.15)^b^	2.49 (1.39)^b^	** < .001**	2.34 (1.26)	2.26 (1.36)	2.15 (1.23)	.322	1.90 (1.14)	1.96 (1.29)	1.91 (1.15)	.927
Have been strangled	Ever been strangled^i^	56.9% (2675)	42.8% (1145)^a^	60.5% (1388)^b^	78.5% (103)^c^	** < .001**	50% (987)^a^	65.8% (73)^b^	67.7% (65)^b^	** < .001**	57.3% (1050)^a^	60% (33)^a^	80.1% (241)^b^	** < .001**
	Strangled during last sex^i^	16.2% (764)	23.9% (255)^a^	34.4% (440)^b^	53.1% (69)^c^	** < .001**	23% (211)	27.9% (19)	30.2% (19)	.306	33% (319)	36.7% (11)	37.8% (84)	.364
	Frequency	5.57 (5.87)	4.84 (5.28)^a^	6.19 (6.33)^b^	6.32 (5.83)^b^	** < .001**	4.74 (5.26)	5.35 (5.20)	5.93 (5.61)	.187	5.76 (6.06)^a^	4.14 (4.37)^a^	7.62 (6.95)^b^	** < .001**
	Very well-known partner	4.09 (1.6)	3.88 (1.17)^a^	4.32 (1.05)^b^	3.87 (1.34)^a^	** < .001**	3.86 (1.17)	4.02 (1.17)	3.98 (1.17)	.456	4.27 (1.10)^a^	4.46 (0.74)^a,b^	4.48 (0.92)^b^	**.017**
	Enjoyed being strangled	3.54 (1.26)	3.39 (1.19)^a^	3.70 (1.26)^b^	3.82 (1.16)^b^	** < .001**	3.34 (1.20)	3.70 (1.20)	3.61 (1.01)	.024*	3.60 (1.27)^a^	3.86 (1.08)^a,b^	4.03 (1.22)^b^	** < .001**
	Partner enjoyed doing it	3.73 (1.07)	3.59 (1.06)^a^	3.88 (1.01)^b^	3.86 (1.14)^b^	** < .001**	3.55 (1.07)	3.84 (0.92)	3.88 (0.93)	.011*	3.84 (1.01)	3.96 (1.14)	4.02 (1.00)	.058
	Wanted to be strangled	3.56 (1.26)	3.43 (1.20)^a^	3.72 (1.25)^b^	3.66 (1.23)^a,b^	** < .001**	3.36 (1.20)^a^	3.86 (1.12)^b^	3.86 (1.09)^b^	** < .001**	3.61 (1.27)^a^	3.86 (1.30)^a,b^	4.09 (1.14)^b^	** < .001**
	Pressure on the neck	3.83 (1.54)	3.95 (1.55)^a^	3.76 (1.51)^b^	4.40 (1.55)^c^	** < .001**	3.91 (1.56)	4.21 (1.33)	4.21 (1.47)	.135	3.68 (1.47)^a^	4.11 (1.34)^a,b^	3.97 (1.58)^b^	**.016**
	+ ve consequences	1.32 (1.33)	1.35 (1.27)	1.31 (1.36)	1.65 (1.44)	**.023**	1.19 (1.29)	1.46 (1.45)	1.71 (1.50)	.102	1.19 (1.29)^a^	1.46 (1.45)^a,b^	1.71 (1.50)^b^	** < .001**
	− ve consequences	0.32 (0.88)	0.30 (0.86)	0.33 (0.88)	0.60 (1.30)	**.002**	0.31 (0.89)	0.16 (0.57)	0.37 (0.86)	.359	0.27 (0.79)^a^	0.11 (0.42)^a^	0.57 (1.21)^b^	** < .001**
Have strangled partners	Ever strangled a partner^i^	50.5% (2374)	59.4% (1323)^a^	40% (917)^b^	74% (134)^c^	** < .001**	59% (1165)	56.8% (63)	69.8% (67)	.093	37.9% (694)^a^	54.5% (30)^b^	50.8% (153)^b^	** < .001**
	Strangling during last sex^i^	14.8% (696)	36.4% (445)^a^	23.1% (194)^b^	48.7% (57)^c^	** < .001**	36.6% (392)	25.9% (15)	42.4% (28)	.147	21.9% (139)	37.9% (11)	27.7% (39)	.058
	Frequency	5.07 (5.54)	5.69 (5.97)^a^	4.31 (4.97)^b^	5.58 (5.30)^a,b^	** < .001**	5.66 (5.94)	4.84 (5.63)	6.57 (6.22)	.285	4.04 (4.67)^a^	3.37 (4.08)^a,b^	5.51 (6.04)^b^	**.005**
	Very well-known partner	4.13 (1.11)	4.11 (1.09)	4.22 (1.09)	3.98 (1.17)	.022*	4.11 (1.08)	4.21 (1.12)	4.07 (1.18)	.727	4.14 (1.14)^a^	4.48 (0.89)^a,b^	4.43 (0.88)^b^	**.008**
	Enjoyed doing it	3.50 (1.12)	3.59 (1.07)^a^	3.39 (1.17)^b^	3.79 (1.03)^a^	** < .001**	3.58 (1.07)	3.54 (1.08)	3.84 (1.04)	.177	3.28 (1.17)^a^	4.04 (0.94)^b^	3.65 (1.16)^b^	** < .001**
	Partner enjoyed it	3.88 (1.09)	3.97 (1.02)	3.86 (1.10)	3.90 (1.17)	.078	3.96 (1.00)	4.07 (1.04)	3.98 (1.16)	.702	3.79 (1.10)^a^	4.33 (0.92)^b^	4.04 (1.11)^b^	**.004**
	Wanted to strangle them	3.42 (1.13)	3.47 (1.10)^a,b^	3.38 (1.15)^a^	3.71 (1.14)^b^	**.008**	3.45 (1.09)	3.55 (1.16)	3.57 (1.23)	.58	3.31 (1.14)	3.78 (1.09)	3.51 (1.20)	.033*
	Pressure on the neck	3.45 (1.64)	3.76 (1.65)^a^	3.06 (1.54)^b^	3.97 (1.73)^a^	** < .001**	3.72 (1.64)	3.62 (1.69)	4.13 1.66)	.146	2.98 (1.51)^a^	3.93 (1.49)^b^	3.26 (1.63)^a,b^	**.002**
	+ ve consequences	1.36 (1.32)	1.50 (1.32)^a^	1.20 (1.32)^b^	1.63 (1.29)^a^	** < .001**	1.48 (1.31)	1.46 (1.44)	1.70 (1.19)	.427	1.10 (1.24)^a^	1.26 (1.29)^a,b^	1.54 (1.51)^b^	**.002**
	-ve consequences	0.20 (0.74)	0.27 (0.94)^a^	0.34 (0.73)^b^	0.22 (0.79)^a^	** < .001**	0.27 (0.89)	0.09 (0.35)	0.26 (0.79)	.314	0.12 (0.46)	0.11 (0.42)	0.19 (0.67)	.350

Bolded text indicates significant difference between groups at *p* < .05

*TGD* trans and gender diverse participants; ^i^ indicates prevalence recorded as % yes (N) and differences tested using chi-square; ^a,b,c^ matching letters across a row indicate no significant differences between two groups; *indicates significant main effect but no differences between groups when applying post hoc multiple comparisons using Bonferroni adjustments

Hierarchical multiple linear regressions were used to assess factors associated with perceptions of being choked (Model 1) and choking (Model 2) sexual partners. These were tested in two regressions with ever having been choked (Model 1) or choking a partner (Model 2) and ever seeing choking in pornography as predictors in Step 1. Step 2 added perceptions that sexual choking can be safe, that it is an expected part of sex, and whether being choked (Model 1) or choking a partner (Model 2) had been discussed with friends. All predictors in both models showed no collinearity (VIF’s < 1.5). All binary correlations with predictors were significant at *p* < 0.001.

## Results

### Strangulation/Choking Awareness

Participants most commonly reported first becoming aware of choking during sex in adolescence at 16–18 years (29%), and early adulthood, 19–21 (24.1%). This varied considerably, with 8.8% of participants reporting that they became aware of sexual choking at the age of 15 or younger, and a proportion (9.4%) reported being unsure when they first learned about it. Few participants (4.9%) indicated they had never heard of sexual choking before the survey. Pornography was the most common avenue by which people reported first hearing about choking during sex (34.8%), followed by discussions with friends (11.5%). Overall, participants had been exposed to information or depictions of sexual choking through various sources, which were primarily via pornography (61.3%), movies (40.3%), friends (31.9%), social media (31.3%), and discussions with potential partners (29.2%). See supplementary material, Table S1, for all ways in which participants had been exposed to sexual choking and proportions within gender.

### Prevalence of Sexual Choking

Overall, more than half (56.9%) of participants reported ever being choked, 50.5% reported ever choking a partner, and 43.9% reported participating in both. On average, participants reported they had been choked 5.6 times, by 3.2 partners, although they were limited in selection to “20 or more” of both. Further, those who had choked partners reported choking someone an average of 5.1 times across 3.3 different partners. However, 22% of participants who had been choked and choked a partner reported only ever engaging in it once before. Type of choking was most commonly reported as one-handed at the last event by 75.5% of people who were choked and 73.5% of participants who choked a partner. Two handed choking was next most common, reported by 20.9% of people choked and 21.3% of chokers. Choking by a belt, rope, tie, or string was reported among 3.4% of those choked and 5.1% of those choking partners. Other choking methods were indicated by 0.2% of both groups. Pressure on the neck was most often reported at a level of 4 out of 7 for both groups. However, 7.6% of people who were choked and 13.1% who choked a partner reported “just resting” pressure at the last event (see supplementary material). Higher levels of pressure on the neck were weakly correlated with more previous experiences of being choked, *r*(2385) = 0.19, *p* < 0.001, and choking someone else, *r*(2076) = 0.20, *p* < 0.001.

Participants reported that the first time they were choked (31.2%) and/or choked a partner (29.9%) most often occurred between the ages of 19–21. Participants most commonly reported first engaging in choking between the ages of 19–21 across all genders and for both being choked or choking a partner. Generally, participants who had been choked neither agreed nor disagreed that being choked (*M* = *3.05, SD* = 1.26) was a common sexual experience for them. Those who had choked partners felt similarly about its commonness (*M* = *3.02, SD* = 1.27).

The proportion of ever having been choked or choking a partner was high across genders. Trans and gender diverse participants were more likely to have ever been choked (78%) followed by women (60.5%), and men (42.8%), *χ*^2^(2, 4702) = 74.18, *p* < 0.001. For those who had ever choked their partners, trans and gender diverse participants were most likely to participate (74%), followed by men (59.4%) and women (40%) *χ*^2^(2, 4702) = 211.69, *p* < 0.001. The same gendered patterns were found for participation in sexual choking at their last sexual experience, see Table 2.

While experiences of sexual choking showed differences across genders, these results were not consistent when comparing across sexuality within men and women, respectively. Among men, differences across sexuality were only found for those who had been choked. Specifically, gay and bisexual men were more likely to have ever been choked than straight men, *χ*^2^(2, 2182) = 20.98, *p* < 0.001. Further, bisexual men were more likely enjoy being choked, want to be choked, and perceive that their partner enjoyed choking them (see Table 2 and supplementary material, Table S2, for *F*-tests and effect sizes).

Among women, differences across sexuality were found for almost all comparisons for both being choked and choking partners. Bisexual women were more likely than straight women to have ever been choked (*χ*^2^(2, 2187) = 55.87, *p* < 0.001) or have choked a partner (*χ*^2^(2, 2187) = 22.89, *p* < 0.001) and have engaged in choking more often, held greater positive general perceptions of choking, were more likely to know their partner well, and have more positive and negative consequences from being choked (see Table 2 and supplementary material, Table S2). Lesbian women were also more likely than straight women to have ever choked a partner, know that partner well, enjoy choking their partners, perceive their partner enjoyed being choked, and to exert more pressure on the neck when engaging in choking. Bisexual women experienced significantly more negative consequences than lesbian women when being choked, but we found no other differences between bisexual and lesbian women.

### Consent

**Fig. 1:**
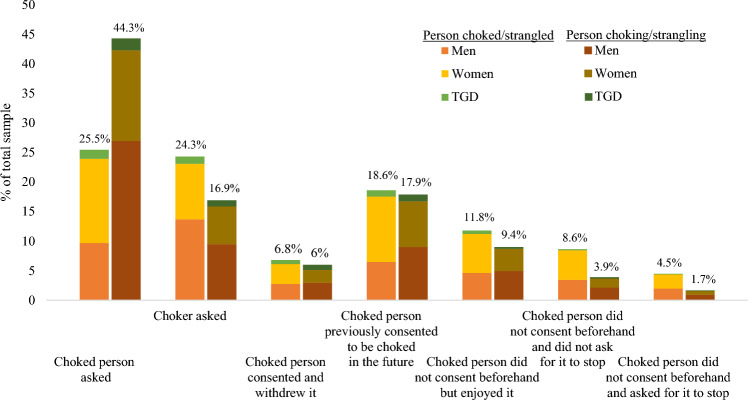
Consent at the last time sexual choking/strangulation event. *Note*: Stacked bar chart represents proportions of the total sample and compares reports of consent given by choked/strangled persons and consent received by choker/stranglers. TGD = transgender and gender diverse participants

Frequencies of the type of consent given by participants the last time sexual choking occurred are presented in Fig. 1 comparing type of participation, split by gender. Overall, participants who had choked partners reported that their partners played an active role in consent more often (79.1%; asking to be choked, agreeing to be choked, or withdrawing previous consent) than those who were choked (56.6%). Participants who were choked more frequently identified that consent was not given beforehand (24.9%) compared to those who had choked partners (15%). Lastly, participants similarly (18.6% of participants choked; 17.9% of participants who choked a partner) reported that consent was negotiated during a previous sexual encounter where the person being choked had given consent to be choked in the future, rather than negotiating or renewing consent during the last event.

Comparisons of proportions across gender on each type of consent showed significant differences between groups on how, or whether, consent was obtained, *χ*^2^(1, 12) = 83.45, *p* < 0.001. Among participants who were choked, women (27.4%) were more likely than men (22.7%), but not trans and gender diverse participants (28.6%) to report that they asked to be choked. Similarly, women (21.2%) were more likely than men (15.2%), but not trans and gender diverse participants (20%) to report that they had given consent during a previous sexual experience. Men (32.1%) were more likely than women (18.1%) and trans and gender diverse participants (22.9%) to report that they agreed to be choked when they were asked by their partner. Trans and gender diverse participants (12.1%) were more likely to report withdrawing consent compared to men (6.5%) and women (6.4%). Lastly, women (9.6%) were more likely than trans and gender diverse participants (3.6%), but not men (8%), to report that they “did not consent, but did not ask or motion for them to stop.” No differences were found for other options where consent was not given beforehand.

Comparisons of proportions across gender on each type of consent among participants who had choked sexual partners were also significant, *χ*^2^(1, 12) = 41.27, *p* < 0.001. Men (47.8%) were more likely than women (40.4%) and trans and gender diverse participants (35.1%) to report that their partner asked to be choked by them. Women were more likely than men to report their partner gave consent to be choked during a previous sexual encounter (16%) but not trans and gender diverse participants (19.8%). Trans and gender diverse participants (14.3%) were most likely to report that their partner withdrew their consent over men (5.2%) and women (5.8%). No other differences were shown across gender variables, see supplementary material, Table S4.

### Positive Perceptions of Sexual Choking

In Model 1, Step 1 showed that age, seeing choking in pornography, and ever previously being sexually choked were found to jointly explain 31.3% of the variance in positive perceptions of being choked, *R*^2^ = 0.32,* F*(3, 4468) = 707.62, *p* < 0.001. Agreement ratings that sexual choking is an expected part of sex, can be done safely, and whether choking had been discussed with friends, jointly contributed a significant increment of 19.3% variance explained when added to the model in Step 2, *R*^2^_change_ = 0.19, *F*_change_ (3, 4466) = 582.84, *p* < 0.001. All predictors accounted for 50.7% of variance in positive perceptions of being choked, adj. *R*^2^ = 0.51, *F*(5, 4466) = 917.02, *p* < 0.001.

In Model 2, seeing choking in pornography, and having ever choked a partner during sex were found to jointly explain 33.1% of the variance in positive perceptions of choking a partner, *R*^2^ = 0.34, *F*(2, 4465) = 1103.36, *p* < 0.001. Agreement ratings that sexual choking is an expected part of sex, can be done safely, and whether choking partners had been discussed with friends, jointly contributed a significant increment of 20.2% variance explained when added to the model, *R*^2^_change_ = 0.20, *F*_change_(3, 4462) = 643.73, *p* < 0.001. All predictors accounted for 53.3% of variance in positive perceptions of choking a partner, adj. *R*^2^ = 0.53, *F*(5, 4462) = 1018.17, *p* < 0.001.

Across both models, views that choking was an expected part of sex, could be done safely, and was discussed with peers, each showed significant unique contributions to perceptions of sexual choking (see Table 3). However, having seen choking depicted in pornography did not contribute to positive perceptions of being choked at Step 2 of Model 1, but still weakly predicted positive perceptions of choking a partner in Model 2 when all other predictors were entered.

**Table 3:** Model 1: Hierarchical multiple regression results for the prediction of positive perceptions of being choked/strangled (Model 1) or choking/strangling a partner (Model 2)

		*β*	95% CI for *β*		*B*	*p*	*r*^2^
			LL	UL			
Model 1	*Step 1*						
	Seen in porn	.06	.07	.17	.12	** < .001**	.059
	Ever being choked	.55	1.04	1.14	1.09	**< .001**	.540
	*Step 2*						
	Seen in porn	.004	− .04	.05	.01	.700	.004
	Ever being choked	.33	.60	.69	.65	**< .001**	.283
	Choking as expected	.26	.19	.22	.21	**< .001**	.251
	Choking can be safe	.35	.25	.29	.27	**< .001**	.311
	Peer discussion of being choked	.10	.12	.19	.15	**< .001**	.089
Model 2	*Step 1*						
	Seen in porn	.07	.09	.19	.14	**< .001**	.070
	Ever choked a partner	.56	1.02	1.11	1.06	**< .001**	.525
	*Step 2*						
	Seen in porn	.02	.001	.08	.04	**.044**	.022
	Ever choked a partner	.32	.57	.66	.62	**< .001**	.274
	Strangling as expected	.30	.21	.25	.23	**< .001**	.278
	Strangling can be safe	.33	.23	.26	.24	**< .001**	.289
	Peer discussion of choking	.10	.12	.19	.16	**< .001**	.092

Bolded text indicates significance *p* < .05

## Discussion

Consistent with US research (Herbenick et al., 2021, 2022a) the findings from this national sample of sexually active young adults shows that exposure and awareness of sexual strangulation among young Australian adults is widespread and is a sexual behavior that has become mainstream—engaged in by cis-men, cis-women, and people who identify as trans and gender diverse. Across the sample, women were more likely to ever have been strangled than men, and men were more likely to have ever strangled partners than women during sex. However, people identifying as trans and gender diverse were most likely than either men or women to engage in both, with approximately three-quarters of this group agreeing that they had ever engaged in it, and approximately half engaging in it the last time they had sex. The frequency that strangulation occurred during sex was also gendered. Women and trans and gender diverse participants reported strangulation happening more often during sex than men. Men reported strangling partners more often during sex than women, although there were no differences across trans and gender diverse participants. These results point to gendered sexual scripts within sexual strangulation, often modeled by pornography, where men are primarily aggressors targeting those with less social power (Bridges et al., 2016; Sun et al., 2017).

Despite the support for sexual scripts, these data also identify that sexual strangulation is not as clearly defined along gender lines, with more than one-third of straight cis-women identifying that they had ever strangled a partner, and over half of straight cis-men ever being strangled. Moreover, sexuality appeared to moderate the gendered patterns among men and women. While straight, gay, and bisexual men showed few differences in their engagement, enjoyment, and pressure engaging in strangulation activities, women on the other hand did show differences. In particular, bisexual women reported the most engagement and enjoyment from being strangled or strangling partners, showing greater engagement, enjoyment, and pressure exerted on the neck, compared to straight women. Kink activities have been found to be commonly engaged in among lesbian and bisexual women, with younger lesbian and bisexual women found to more likely participate in asphyxiation/breath play (Pavanello Decaro et al., 2024; Tomassilli et al., 2009). For some people who are bisexual, pansexual, or queer, these types of kink activities can be an avenue to explore gender identity and sexual orientation, with some people using the activities and kink communities to help them heal from trauma (Sprott & Benoit Hadcock, 2018). This makes clear that care needs to be taken to avoid the stigmatization of these increasingly common practices to not further harm already marginalized groups, and we recognize the role that articles such as this play in the discourse around the use of language to silence and shame (e.g., “breath play” vs “asphyxiation” and “choking” vs “strangulation”; see Cardoso, 2022). However, strangulation in the course of erotic asphyxiation is the leading cause of death in BDSM play (bondage and discipline, dominance and submission, sadism, and masochism; Schori et al., 2022) and the dangers of engaging in strangulation are well-established in scientific and medical research (Bichard et al., 2021). Research suggests that the dangers are unlikely to be known or appreciated by the general population more broadly (Sharman et al., 2024).

Negotiations of consent largely showed that the last time strangulation occurred during sex, consent of the person being strangled appeared to be actively agreed to during the sexual experience (either verbally or through gestures). However, for many others, consent to be strangled was not discussed directly before or during the sexual experience. This was particularly clear among participants who were strangled, compared to those strangling, who more frequently identified that even if they enjoyed it, they did not ask for, or discuss, strangulation before it occurred during sex. In this way, it may be seen as an act that is part of sex, where consent to strangulation is not needed when there is consent to sex (Beres, 2020; Jozkowski, 2014). However, this line is clearly blurred when strangulation is unwanted, particularly for those being strangled. Taking on submissive roles may hinder them from voicing concerns about being strangled or saying no, potentially going along with the act despite feeling uncomfortable or scared, or worse, being physically unable to voice or gesture for a removal of consent (Herbenick et al., 2022b; Rossen et al., 1943).

This lack of specified consent is likely to be linked to ideas that sexual strangulation is safe and enjoyable, as found by Wright et al., (2023a). Findings in this study complement this previous research and supported our hypothesis showing that, after controlling for exposure to strangulation in pornography and previous experiences of sexual strangulation, views that it can be done safely and social factors surrounding strangulation contribute to participants’ positive perceptions of it. Specifically, whether it is normative and expected as part of sex and whether they have discussed strangulation with their peers. However, despite pornography being a primary source for participants to learn about sexual strangulation, having seen strangulation as part of pornography only weakly predicted positive perceptions of strangling a partner, but not of being strangled, net of other factors. These results indicate that pornography may play a key role in introducing people to strangulation during sex, but their experiences and expectations of safety help to shape their attitudes and perceptions. Similar to Wright et al. (2023a, 2023b), future research should explore whether exposure to depictions of strangulation in pornography may predict positive perceptions of strangulation during sex via pathways such as normative beliefs and perceived safety.

Overall, perceptions of sexual strangulation as normal and safe were significant contributors to viewing it in a positive light after adjusting for exposure to strangulation in pornography and previous participation, and that these contributors were comparable toward strangling a partner or being strangled. Development of sexual education interventions with younger people to improve understanding of the array of potential harms and changing normative expectations around sexual strangulation, and misinformation from pornography and other sources (Herbenick et al., 2023b, 2023c), may help to reduce positive perceptions and future engagement (Crabbe & Flood, 2021; Maas et al., 2022). The most frequent age of first learning about strangulation was in adolescence between 16 and 18 years, so education strategies targeted at this age group may be appropriate to reduce risk of harm.

### Limitations and Future Research

This study is the first to examine the national prevalence of sexual strangulation among adults 18–35 years in Australia. However, there were limitations to this research. Firstly, there were limitations in the definition of strangulation/choking provided to participants by excluding blood choking. The definition was provided to participants after they had answered whether they had prior experiences being choked or choking a partner during sex, consequently responses to this first set of choking questions may have captured both blood and breath choking, and we are unable to differentiate the two. The clarification in the definition following the initial choking questions may have impacted how some participants who had experiences of, or knew about, blood choking responded to questions thereafter. This, coupled with stigma about some forms of sexual practice (Schuerwegen et al., 2022), may have affected how people responded to questions such as how many times they had been choked/choked a partner during sex. Further, we did not provide a definition of sex for participants, so we are unable to identify what kind of sexual practices people engage in alongside strangulation, or whether the strangulation is, by itself considered a sexual experience by some.

Secondly, this cross-sectional survey was not able to capture the nuance of qualitative studies, including the complexities of smaller social groups. While we were able to include transgender and gender diverse people in our analyses, due to the heterogeneity of this combined group we were not able to provide more detailed analysis by sexual orientation. Given that the results of this cohort revealed a high rate of participation in sexual strangulation and greater pressure on the neck reported by trans and gender diverse participants who had been strangled, it is important that we gain further insight into how and why strangulation is more common among this group in future qualitative and mixed methods research. Additionally, we were not able to explore more thoroughly the differences in pressure (such as a hand “just resting”) and how strangulation is perceived, particularly among the 7.5% of participants who reported this pressure during their last experience. Qualitative exploration will provide greater detail on how perceived pressure and placement on the neck changes feelings of desirability.

Thirdly, consequences reported in this study were low compared to previous research within and outside of Australia (Herbenick et al., 2022a; Sharman et al., 2024). Because many prior questions were focused on the last time a person had been strangled, participants may have been more likely to provide consequences referencing their last sexual strangulation experience rather than consequences that had “ever” occurred. Further, although we provided several different options, they were not exhaustive and may have missed key positive/pleasurable or negative/physical outcomes relating to sexual strangulation. Despite this we still found differences in consequences between groups, particularly among bisexual women who experienced both more positive and more negative consequences than straight women. Given recent findings of deficits in cognitive functioning following recent experiences of sexual strangulation (Huibregtse et al., 2022), future research on this topic should explore the frequency of strangulation in more nuanced ways (e.g., number of times strangled in a single sexual experience), and consequences that occur longitudinally, including those that may not adequately be captured in cross-sectional survey research such as memory deficits. Further, we were unable to capture strangulation during other intimate activities that did not involve or lead to sex, such as kissing. This may impact people who are yet to engage in sex and who may be younger, yet still experiencing consequences related to strangulation. Longitudinal investigations such as this will give us a better picture of how strangulation practices and associated harms are changing over time.

Lastly, most of the analyses of behaviors, pressure and perceptions related to strangulation showed small effect sizes when compared between groups. This indicates that the size of the differences between groups were less practically significant and likely found because of the large sample size. These small effect sizes limit the strength of conclusions based on differences between groups. However, they highlight the overall finding that regardless of gender or sexual orientation, sexual strangulation is prevalent in Australia among 18–35-year-olds.

### Conclusion

Our findings reveal that, in Australia, sexual strangulation has become a mainstream sexual behavior that is commonly seen in media such as pornography and movies and discussed among friends. It is engaged in by more than half of men, women, and trans and gender diverse people aged 18–35 who have previously had sex. Largely, strangulation during sex is viewed positively and this was predicted by the perception that it can be safe and is an expected and social normative behavior. These perceptions of safety are at odds with the numerous and potentially significant harms that strangulation can cause and worryingly, a large proportion of participants thought prior consent for sexual strangulation was an acceptable form of consent for future choking activities. Here, there was a general presumption that consent could be provided once, and no further consent or negotiation at subsequent events would be required. These results indicate the need for developing strong sexual health education around consent, harms, and normative expectations around sexual strangulation in Australia.

## Supplementary Information

Below is the link to the electronic supplementary material.

Supplementary file 1 (DOCX 43 kb)

## Data Availability

Readers interested in the data for the current study are invited to contact the corresponding author.
