# Correction: Prevalence of Sexual Strangulation/Choking Among Australian 18–35 Year-Olds

**DOI:** 10.1007/s10508-024-03014-0

**Published:** 2024-09-25

**Authors:** Leah S. Sharman, Robin Fitzgerald, Heather Douglas

**Affiliations:** 1 Melbourne Law School, Faculty of Law, University of Melbourne, Melbourne, VIC 3010 Australia; 2 Faculty of Humanities and Social Science, University of Queensland, Brisbane, Australia

**Correction to Archives of Sexual Behavior** 10.1007/s10508-024-02937-y

The frequency stated for both being choked and choking in the first sentence of the Prevalence of Sexual Choking section was incorrect in the article as originally published and should have been 43.9%.

The original article has been corrected.

