# Supplementary material for: Prevalence of Sexual Strangulation/Choking Among Australian 18–35 Year-Olds
Source: Arch Sex Behav. 2024 Jul 3;54(2):465–80. doi: 10.1007/s10508-024-02937-y (PMC11836212; doi:10.1007/s10508-024-02937-y)
Supplement: Supplementary file 1 — (DOCX 43 kb) [file 10508_2024_2937_MOESM1_ESM.docx]

Table S1. Ways in which participants had learned or heard about sexual strangulation.

|  | First learned/heard % (N) | | | | Ever from other sources %* | | | | |
| --- | --- | --- | --- | --- | --- | --- | --- | --- | --- |
|  | Total % (N) | Men | Women | TGD | Total % (N) | Men | Women | TGD |  |
| Pornography | 34.8% (1636) | 50.3% | 20.1% | 30.4% | 61.3% (2880) | 71.4% | 51.5% | 60.2% |  |
| Erotica | 2.8% (133) | 2.1% | 3.2% | 7.2% | 17.7% (833) | 18.1% | 16% | 33.7% |  |
| Magazine articles | 1.5% (70) | 1.4% | 1.3% | 5.5% | 8.7% (409) | 9% | 7.6% | 19.3% |  |
| Books | 3% (140) | 1.8% | 3.7% | 8.8% | 18.8% (883) | 13.3% | 22.8% | 35.4% |  |
| Movies | 9.7% (456) | 7% | 12.3% | 11% | 40.3% (1894) | 37.5% | 42.3% | 48.6% |  |
| Social media | 6.4% (302) | 5.4% | 7.2% | 8.3% | 31.3% (1473) | 30.7% | 31.6% | 35.9% |  |
| Discussion with a (potential) sexual partner in person | 9.2% (434) | 8.9% | 11.6% | -^^^ | 29.2% (1375) | 27.8% | 30.3% | 33.7% |  |
| Discussion with a (potential) sexual partner online | 1% (49) | 1.1% | 0.9% | -^^^ | 11.7% (548) | 11.8% | 10.6% | 22.7% |  |
| Discussion(s) with friends | 11.5% (540) | 8.1% | 17.6% | 7.2% | 31.9% (1501) | 26.4% | 37.2% | 67.4% |  |
| Trying it without discussion | 3.7% (175) | 1.6% | 6.7% | -^^^ | 8.9% (420) | 5.4% | 12.3% | 8.8% |  |
| Dating app or website | 0.4% (19) | -^^^ | -^^^ | -^^^ | 6.7% (313) | 7.4% | 5.7% | 9.9% |  |
| Other | 0.9% (41) | 0.7% | 1% | -^^^ | 1.3% (59) | 1.1% | 1.3% | -^^^ |  |
| Unsure | 10.1% (474) | 7.4% | 13.2% | -^^^ | N/A | - | - | - |  |
| I have never learned or heard about this | 3.9% (183) | 3% | 5% | -^^^ | N/A | - | - | - |  |

TGD = trans and gender diverse participants

*More than one answer could be selected so totals are > 100%;

^ percentages with Ns <10 not provided.

Table S2. Mean differences and significance values of sexual strangulation and related behaviours, consequences, and acceptance across gender and sexual orientation.

|  | Total (mean difference, significance) | | | | | | | Men (mean difference, significance) | | | | | | | Women (mean difference, significance) | | | | | | |
| --- | --- | --- | --- | --- | --- | --- | --- | --- | --- | --- | --- | --- | --- | --- | --- | --- | --- | --- | --- | --- | --- |
|  | *F* | df, error | $\eta$_p_^2^ | Man- Woman | Man - TGD | Woman - TGD | *F* | | df, error | $\eta$_p_^2^ | Straight - Gay | Straight - Bi | Gay - Bi | *F* | | df, error | $\eta$_p_^2^ | Straight - Lesbian | Straight - Bi | Lesbian - Bi |  |
| Perception of being strangled | 29.093 | 2, 4558 | .013 | -0.041, .651 | **-.656, <.001** | **-0.615, <.001** | 3.697 | | 2, 2132 | .003 | -0.16, .235 | -0.21, .094 | -0.05, 1.00 | 35.548 | | 2, 2121 | .032 | -0.20, .423 | **-0.53, <.001** | -0.33, .082 |  |
| Perception of strangling | 95.430 | 2, 4558 | .040 | **0.412, <.001** | **-0.302, .001** | **-0.714 <.001** | 0.121 | | 2, 2132 | .000 | -0.04, 1.00 | -0.03, 1.00 | 0.01, 1.00 | 26.238 | | 2, 2121 | .024 | **-0.35, .015** | **-0.39, <.001** | -0.04, .1.00 |  |
| Choking is safe | 4.196 | 2, 4528 | .002 | 0.00, 1.00 | **-0.29, .014** | **-0.29, .013** | 3.826 | | 2, 2104 | .004 | **-0.34, .018** | -0.06, 1.00 | 0.28, .322 | 31.047 | | 2, 2114 | .029 | **-0.52, .014** | **-0.63, <.001** | -0.10, 1.00 |  |
| Choking is expected | 71.934 | 2, 4528 | .031 | **0.41, <.001** | -0.17, .218 | **-0.59, <.001** | 1.133 | | 2, 2104 | .001 | 0.08, 1.00 | 0.19, .481 | 0.11, 1.00 | 0.076 | | 2, 2114 | .000 | -0.06. 1.00 | -0.01, 1.00 | 0.05, 1.00 |  |
| Recipient frequency | 15.411 | 2, 2351 | .013 | **-1.34, <.001** | **-1.48, .022** | 0.14, 1.00 | 1.679 | | 2, 987 | .003 | -0.61, 1.00 | -1.19, .295 | -0.58, 1.00 | 9.355 | | 2, 1159 | .016 | 1.62, .524 | **-1.86, <.001** | **03.47, .016** |  |
| Knew the actor very well | 45.449 | 2, 2351 | .037 | **-0.44, <.001** | 0.02, 1.00 | **0.45, <.001** | 0.786 | | 2, 987 | .002 | -0.16, .906 | -0.12, 1.00 | 0.03, 1.00 | 4.100 | | 2, 1159 | .007 | -0.20, .982 | -0.02, 1.00 | 0.02, 1.00 |  |
| I enjoyed being choked | 20.278 | 2, 2351 | .017 | **-0.31, <.001** | **-0.43, <.001** | 0.12, .892 | 3.764 | | 2, 987 | .008 | -0.35, .068 | -0.27, .290 | .08, 1.00 | 10.592 | | 2, 1159 | .018 | -0.25, .881 | **-0.43, <.001** | -0.17, 1.00 |  |
| My partner enjoyed choking me | 21.781 | 2, 2351 | .018 | **-0.29, <.001** | -0.27, .098 | 0.02, 1.00 | 4.491 | | 2, 987 | .009 | -0.29, .106 | -0.33, .072 | -0.04, 1.00 | 2.847 | | 2, 1159 | .005 | -0.12, 1.00 | -0.18, .058 | -0.06, 1.00 |  |
| I wanted to be choked | 15.147 | 2, 2351 | .013 | **-0.29, <.001** | -0.23, .140 | 0.06, 1.00 | 9.237 | | 2, 987 | .018 | **-0.50, .004** | **-0.50, .007** | .00, 1.00 | 13.590 | | 2, 1159 | .023 | -0.25, .890 | **-0.48, <.001** | -0.23, 1.00 |  |
| Pressure experienced | 12.520 | 2, 2351 | .011 | **0.20, .008** | **-0.45, .006** | **-0.64, <.001** | 2.008 | | 2, 987 | .004 | -0.30, .413 | -0.30, .453 | .00, 1.00 | 4.178 | | 2, 1159 | .007 | -0.43, .406 | **-0.29, .030** | 0.14, 1.00 |  |
| Actor frequency | 14.585 | 2, 2038 | .014 | **1.38, <.001** | 0.11, 1.00 | -1.27, .063 | 1.256 | | 2, 1121 | .002 | 0.82, .938 | -0.91, .733 | -1.73, .344 | 5.412 | | 2, 738 | .014 | 0.67, 1.00 | **-1.47, .005** | -2.14, .118 |  |
| How well known was the recipient | 3.808 | 2, 2038 | .004 | -0.11, .079 | 0.12, .726 | 0.24, .085 | 0.319 | | 2, 1121 | .001 | -0.11, 1.00 | .04, 1.00 | 0.15, 1.00 | 4.860 | | 2, 738 | .013 | -0.34, .340 | **-0.29, .015** | 0.05, 1.00 |  |
| I enjoyed choking my partner | 10.862 | 2, 2038 | .011 | **0.20, <.001** | -0.20, .172 | **-0.40, <.001** | .735 | | 2, 1121 | .003 | 0.04, 1.00 | -0.26, .207 | -0.30, .390 | 10.166 | | 2, 738 | .027 | **-0.75, .003** | **-0.37, .002** | -0.38, .351 |  |
| My partner enjoyed being choked | 2.549 | 2, 2038 | .002 | 0.11, .074 | 0.07, 1.00 | -.04, 1.00 | 0.355 | | 2, 1121 | .001 | -0.12, 1.00 | -0.03, 1.00 | 0.09, 1.00 | 5.628 | | 2, 738 | .015 | **-0.55, .033** | **-0.25, .048** | 0.30, .595 |  |
| I wanted to choke them | 4.883 | 2, 2038 | .005 | 0.09, .263 | -0.24, .077 | **-0.33, .008** | 0.544 | | 2, 1121 | .001 | -0.10, 1.00 | -0.12, 1.00 | -0.02, 1.00 | 3.413 | | 2, 738 | .009 | -0.47, .119 | -0.20, .222 | 0.27, .793 |  |
| Pressure given | 48.212 | 2, 2038 | .045 | **0.69, <.001** | -0.22, .484 | **-0.91, <.001** | 1.924 | | 2, 1121 | .003 | 0.10, 1.00 | -0.41, .179 | -0.51, .288 | 6.380 | | 2, 738 | .017 | **-0.95, .005** | -0.29, .148 | 0.66, .122 |  |

Bolded values indicate significant differences between groups using multiple comparisons with Bonferroni corrections;

TGD = trans and gender diverse participants

Table S3. Consequences reported from sexual strangulation

| Consequence | Strangled | Strangler |
| --- | --- | --- |
| Nothing happened | 24.4% | 24.1% |
| Strangled person (SP) enjoyed sex more | 45.1% | 47% |
| Person strangling enjoyed sex more | 33.8% | 35% |
| SP had a more intense orgasm | 23.9% | 28.3% |
| Person strangling had a more intense orgasm | 18.6% | 17.4% |
| SP felt a dizziness they liked | 11.1% | 7.9% |
| SP Dizziness they did NOT like | 3.6% | 3.1% |
| SP Vision changed | 3.5% | 2.7% |
| SP Couldn't breathe | 6% | 2.9% |
| SP Sore throat | 4.3% | 2.3% |
| SP Voice changed or lost their voice | 1.8% | 1.4% |
| SP Headache | 2% | 1.1% |
| SP Marks or bruises around the neck | 4.7% | 2.2% |
| SP Couldn't move or speak | 1.6% | 1% |
| SP Blacked out/became unconscious | 1.3% | 1.2% |
| SP Bloodshot eyes | 1.3% | 0.8% |
| SP Lost control of their bladder | 1% | 0.9% |
| SP Lost control of their bowels | 0.6% | 0.5% |
| SP Unsure | - | 2.3% |
| Other | 0.6% | 0.1% |
| Panic attack/anxiety/scared | * | - |
| invisible bruising around the neck | * | - |
| Nose bleed | * | - |
| difficulty breathing | * | - |
| Uncomfortable | * | - |

More than one answer could be selected so totals are > 100%;

*percentages with Ns <10 not provided.

**Figure S1.**

*Reported Pressure on the Neck at the Last Time Choking/Strangulation Event.*

Table S4. Proportions of whether consent was given or received the last time participants engaged in sexual strangulation

|  | Strangled person | | | | Strangler | | | |
| --- | --- | --- | --- | --- | --- | --- | --- | --- |
|  | Total % (N) | Men | Women | TGD | Total % (N) | Men | Women | TGD |
| Strangled person asked to be choked | 25.5% (661) | 22.7% | 27.4% | 28.6% | 44.3% (1014) | 47.8% | 40.4% | 35.1% |
| Strangler asked to choke strangled person | 24.3% (631) | 32.1% | 18.1% | 22.9% | 16.9% (387) | 16.8% | 16.8% | 18.3% |
| Strangled person consented and withdrew it | 6.8% (176) | 6.5% | 6.4% | 12.1% | 6% (137) | 5.2% | 5.8% | 15.3% |
| Strangled person previously consented to be choked in the future | 18.6% (483) | 15.2% | 21.2% | 20% | 17.9% (409) | 16.0% | 20.4% | 19.8% |
| Strangled person did not consent beforehand but enjoyed it | 11.8% (306) | 10.8% | 12.7% | 10.7% | 9.4% (206) | 8.8% | 9.9% | -^^^ |
| Strangled person did not consent beforehand and did not ask for it to stop | 8.6% (224) | 8.0% | 9.6% | -^^^ | 3.9% (89) | 3.8% | 4.0% | -^^^ |
| Strangled person did not consent beforehand and asked for it to stop | 4.5% (116) | 4.6% | 4.6% | -^^^ | 1.7% (38) | 1.6% | 1.6% | -^^^ |

TGD = trans and gender diverse participants

^ percentages with Ns <10 not provided.
